# An Extensive Targeted Proteomic Analysis of Disease-Related Protein Biomarkers in Urine from Healthy Donors

**DOI:** 10.1371/journal.pone.0063368

**Published:** 2013-05-28

**Authors:** Brian M. Nolen, Lidiya S. Orlichenko, Adele Marrangoni, Liudmila Velikokhatnaya, Denise Prosser, William E. Grizzle, Kevin Ho, Frank J. Jenkins, Dana H. Bovbjerg, Anna E. Lokshin

**Affiliations:** 1 University of Pittsburgh Cancer Institute, Hillman Cancer Center, Pittsburgh, Pennsylvania, United States of America; 2 Department of Pathology, University of Alabama at Birmingham, Birmingham, Alabama, United States of America; 3 Department of Medicine, The Renal-Electrolyte Division, School of Medicine, University of Pittsburgh, Pittsburgh, Pennsylvania, United States of America; 4 Department of Pathology, School of Medicine, University of Pittsburgh, Pittsburgh, Pennsylvania, United States of America; 5 Department of Psychiatry, School of Medicine, University of Pittsburgh, Pittsburgh, Pennsylvania, United States of America; 6 Department of Psychology, Dietrich School of Arts and Sciences, University of Pittsburgh, Pittsburgh, Pennsylvania, United States of America; 7 Department of Behavioral and Community Health Sciences, Graduate School of Public Health, University of Pittsburgh, Pittsburgh, Pennsylvania, United States of America; 8 Department of Medicine, School of Medicine, University of Pittsburgh, Pittsburgh, Pennsylvania, United States of America; 9 Department of Ob/Gyn, School of Medicine, University of Pittsburgh, Pittsburgh, Pennsylvania, United States of America; Wageningen UR Livestock Research, The Netherlands

## Abstract

The analysis of protein biomarkers in urine is expected to lead to advances in a variety of clinical settings. Several characteristics of urine including a low-protein matrix, ease of testing and a demonstrated proteomic stability offer distinct advantages over current widely used biofluids, serum and plasma. Improvements in our understanding of the urine proteome and in methods used in its evaluation will facilitate the clinical development of urinary protein biomarkers. Multiplexed bead-based immunoassays were utilized to evaluate 211 proteins in urines from 103 healthy donors. An additional 25 healthy donors provided serial urine samples over the course of two days in order to assess temporal variation in selected biomarkers. Nearly one-third of the evaluated biomarkers were detected in urine at levels greater than 1ng/ml, representing a diverse panel of proteins with respect to structure, function and biological role. The presence of several biomarkers in urine was confirmed by western blot. Several methods of data normalization were employed to assess impact on biomarker variability. A complex pattern of correlations with urine creatinine, albumin and beta-2-microglobulin was observed indicating the presence of highly specific mechanisms of renal filtration. Further investigation of the urinary protein biomarkers identified in this preliminary study along with a consideration of the underlying proteomic trends suggested by these findings should lead to an improved capability to identify candidate biomarkers for clinical development.

## Introduction

Protein biomarkers represent the myriad aspects of cellular physiology altered in response to disease. The measurement of protein biomarkers through proteomics, immunoassays, immunohistochemistry or various other novel techniques has formed the basis for the development of tools currently utilized in numerous clinical settings. Realized and potential applications include early detection, disease monitoring, prognostication, and evaluation of treatment response. Protein biomarkers have also emerged as important tools within the arena of pharmaceutical development, serving as companion diagnostics to novel therapeutics which aid in patient selection, treatment monitoring, adverse event risk assessment, and the extension of indications for established drugs. Despite the widespread appreciation of the usefulness and potential benefits of protein biomarker use and the considerable attention devoted to biomarker research, progress has been hampered by several factors. The vast majority of protein biomarkers currently in use or under investigation do not represent novel pathological entities, but merely dysregulated aspects of normal physiology. Thus, biomarker development requires extensive preclinical characterization in order to overcome inherent limitations in sensitivity and specificity.

The bulk of protein biomarker research has focused on blood, given its systemic exposure and extensive availability through tissue banks. The analysis of blood, either through the use of serum or plasma, carries with it several inherent limitations which have delayed the development of clinically useful biomarker assays. Foremost among these limitations is the abundant and complex protein repertoire found in blood. Components of the blood matrix, including clotting and other serological factors, carrier proteins, immunoregulatory proteins, and active enzymes all have the capacity to interfere with biomarker measurements. The clotting process itself, employed during the preparation of serum, has been shown to involve enzymatic activity which results in the cleavage of unrelated proteins of interest [1], [2]. The invasive nature of blood testing also limits accessibility to repeated measurements and presents the added cost of minimizing the risk of infection. The use of small bore needles may also lead to endothelial cell activation and the production of analytical artifacts [3]. Urine presents an attractive alternative biofluid for analytical biomarker studies in that the systemic nature of such testing might be preserved while several of the limitations inherent to blood testing could be eliminated. Urine is available in larger quantities than blood through less invasive means, allowing for repeated measurements aimed at patient surveillance or establishment of assay reproducibility. Renal filtration also results in a less complex matrix than that of blood, containing fewer factors known to interfere with biomarker assays [4]. This is supported by studies demonstrating a high stability of urinary proteins reported to be hours at room temperature, days at 4°C, and years at −20°C [5].

Investigations into the clinical applications of urinary proteomics to date have been fruitful. Reported findings have largely focused on the use of urinary protein biomarkers in nephrological and urological disorders, allograft rejection, and prognosis associated with diabetic nephropathy and lupus (reviewed in [6], [7]). However, a number of reports have demonstrated extended applications for urine biomarkers beyond renal disease in settings such as acute pancreatitis [8], obstructive sleep apnea [9], lung cancer [10], and ovarian, breast and pancreatic cancer (reviewed in [11]). Work in this area has been supported by our evolving understanding of the urine proteome. The urine proteome represents the integrated product of glomerular filtration of plasma and protein shedding by cells of the proximal renal tubule, suggestive of both systemic and local contributions. Adachi et al. utilized high performance liquid chromatography coupled to mass spectrometry (HPCL-MS) to identify over 1500 urinary proteins in healthy individuals [12]. A subsequent effort employing capillary electrophoresis coupled to mass spectrometry (CE-MS) identified over 100,000 distinct peptides in the urine proteome with 5000 of those present at high frequency [13]. More recently, Kentsis et al. subjected urines from 12 individuals to extensive fractionation followed by proteomic analysis and identified >2300 unique proteins, included 1000 not described previously [14]. In the current study, we sought to further advance the study of urine proteomics through the use of an extensive array of multiplexed immunoassays targeting a diverse panel of disease-related protein biomarkers in a large group of healthy individuals.

## Materials and Methods

### Human Subjects

Two separate sets of human subjects were utilized in the current study (Table 1). Set I consisted of 103 healthy urine donors. Urines from 75 healthy donors were obtained from Proteogenex Inc. (Culver City, CA). An additional 28 urines obtained from healthy individuals were collected at the University of Alabama at Birmingham (UAB) in support of the Pancreatic SPORE (P20 CA101955). Set II consisted of urine samples collected over three time intervals daily (day, evening, night) over the course of two study days from 25 healthy female donors at the University of Pittsburgh. All subjects were over the age of 18 and provided written informed consent. Pregnant women and subjects with a history of blood borne illness were excluded. All donors were cancer free and free of other major illness at the time of donation. All urine samples in Set I were spot collected at the time of medical visit or donation. Urine samples in Set II were collected over specified time intervals by participants. Urines were frozen at −70°C or −80°C without further processing following collection and remained frozen until testing. Each urine sample was annotated with information regarding age, gender, ethnicity, and smoking history (when available).

**Table 1:** Characteristics of study population.

	Set I (n = 103)	Set II (n = 25)
Age Range (median)	29–70 (57)	25–49 (36)
**Gender**		
Male	40	0
Female	63	25
**Race**		
Caucasian	91	19
Asian/Pacific Islander	7	4
African American	4	2
Unknown	1	0
**Smoking Status**		
Current/Previous Smoker	37	5
Never	37	20
Unknown	29	0

### Ethics Statement

All subjects involved in this study were over the age of 18 and provided written informed consent. Collection of samples at the University of Pittsburgh was performed according to strict protocols approved by the University of Pittsburgh Institutional Review Board. Collection of samples at the University of Alabama at Birmingham (UAB) was performed according to strict protocols approved by the UAB Institutional Review Board for Human Use. Collection of samples by Proteogenix, Inc. was performed according to strict protocols approved by an independent ethics committee at the Russian Oncological Research Center (Moscow, Russia).

### Multiplexed Biomarker Analysis

The xMAP^TM^ bead-based technology (Luminex Corp., Austin, TX) permits multiplexed analysis of multiple analytes in a single sample. An array of 211 bead-based immunoassays targeting a diverse set of protein biomarkers available on the xMAP^TM^ Luminex platform was utilized in this study (Table S1 in File S1). This list of biomarkers was assembled based on a literature review of proteins and families of proteins of interest in all areas of circulating biomarker research. Biomarkers were selected from this list on the basis of suitable immunoassay availability. A total of 40 separate multiplexed assays were utilized. Bead-based immunoassays targeting angiostatin, Bcl-2, CA 15-3, CA 19-9, CA 125, CA 72-4, CD-105, CEA, cytokeratin 19, EGFR, endostatin, EPCAM, ErbB2, fPSA, HSP70, IGFBP-2, kallikrein 10, mammaglobin, MICA, HE4, NSE, oncostatin, PBEF, PSA, thrombospondin, TgII, and SCC were developed by the UPCI Luminex Core Facility according to strict quality control standards [15]. Assays for MMP 1, 2, 3, 7, 8, 12, 13 and TIMP 1-4 were obtained from R&D Systems (Minneapolis, MN). Assays for BLC/CXCL12, granzyme A, granzyme B, INF-β, INF-ω, perforin, sCD137/4-1BB were obtained from BioLegend (San Diego, CA). All other assays were obtained from Merck/Millipore (Durmstadt, Germany). All commercial immunoassays were performed according to manufacturer's protocols while UPCI Luminex Core Facility assays were performed as previously described [16], with the exception that all urine samples were tested undiluted. All biomarker testing was performed immediately upon thawing with no protein concentration or other manipulation. Urines in Set I were tested for the complete panel of 211 biomarkers. Urines in Set II were tested for a subset of 29 cytokines, hormones, and other glycoproteins hypothesized to be responsive to circadian rhythms (IL-1B, IL-6, IL-8, leptin, TNFa, MCP-1, HGF, Insulin, NGF, AGRP, FSH, BDNF, LH, TSH, prolactin, GH, ACTH, CNTF, sE-Selectin, sVCAM-1, sICAM-1, MMP-9, MPO, adiponectin, tPAI-1, eotaxin, IP-10, HE4, CA 15-3).

### Measurement of urine parameters

The total protein content of each urine was measured using the Bradford method (Bio-Rad Life Science Research, Hercules, CA). Urine creatinine (UCr) levels were determined for each urine sample using the Creatinine Parameter Assay Kit (R&D Systems, Minneapolis, MN). The measurement of urine albumin levels was included in the multiplexed biomarker analysis described in the previous section utilizing reagents obtained from Merck/Millipore (Durmstadt, Germany).

### Statistical analysis of data

Each urine sample was analyzed for each multiplex immunoassay using the Bio-Plex suspension array system (Bio-Rad Laboratories, Hercules, CA). For each analyte, 100 beads were analyzed and the median fluorescence intensity was determined. Analysis of experimental data was performed using five-parameter logistic curve fitting to standard analyte values in order to generate observed concentration values for each protein. In an effort to account for individual variation in fluid intake, urine biomarker measurements were initially normalized based on urinary creatinine (UCr) levels by dividing the observed concentration of each urine protein by the UCr level (mg/dL) for each respective sample. Subsequent normalization based on other urine parameters (total protein, albumin, albumin to creatinine ratio, beta-2-microglobulin) was conducted in the same manner. Biomarker distributions among the experimental groups were evaluated using standard statistical methods in order to generate mean, median and %CV values for each set of measurements within each group. All analysis of correlations was performed using the Pearson test of correlation in GraphPad Prism (La Jolla, CA) with a minimum level of significance of p<0.05.

### Antibodies, SDS-PAGE, Western Blotting

Mouse anti-CA125 M11 (Catalog No. 205–485) and anti-HE4 (Catalog No. 3D8) monoclonal antibodies were purchased from Fujirebio Diagnostics, Inc. (Malvern, PA). Mouse anti-Osteopontin N-half (Catalog No. 34E3) antibody was from IBL Co., Ltd (Gunma, Japan). Anti-TTR (Catalog No. H00007276-M01) mouse monoclonal antibody was purchased from Abnova (Taipei, Taiwan). Mouse anti-CA 125 (Catalog No. 10-C02) clone M8072320 monoclonal antibody against the CA125 protein was from Fitzgerald Industries International (Action, MA). Mouse alpha-HRP antibody (Catalog No. 31430) was from Pierce (Thermo Fisher Scientific, Rockford, IL). Sheep anti-human Tamm-Horsfall protein (Catalog No. AB733) was from EMD Millipore (Temecula, CA). Protein samples for Western blotting were obtained from concentrated human urine collected from five healthy donors randomly selected from Set I (Table 1). Protein concentration was determined by Bradford method using a kit from Bio-Rad (Bio-Rad, Hercules, CA). Proteins were separated on 12% SDS-PAGE followed by transfer to polyvinylidene difluoride (PVDF) membranes. SDS-PAGE [17], transfer of proteins to PVDF membranes [18] and Western blotting [19] were as described. Immunodetection of bound antibodies on PVDF membrane was performed using ECL reagents (Amersham Biosciences, Piscataway, NJ). All procedures were carried out according to the manufacturer's instructions. Tamm-Horsfall protein (THP) was assayed as a loading control.

## Results

### Urine Albumin and Total Protein Content

**Figure 1:**
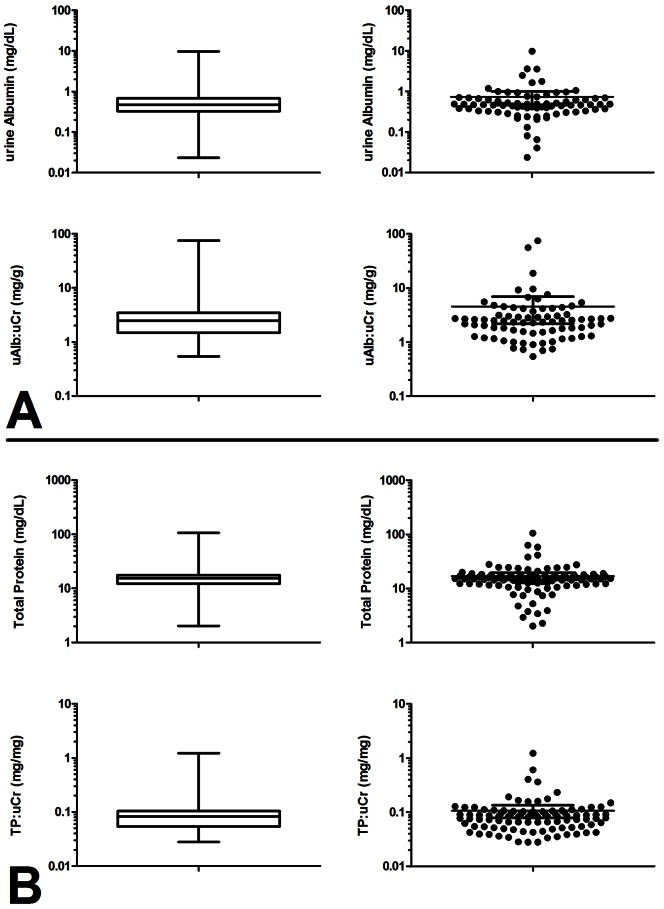
Distribution of urine albumin and total protein levels in healthy donors. A. Box-whisker and scatter plots of urine albumin distributions in urines. (Top panels: urine albumin concentration in mg/dL; Bottom panels: ratio of urine albumin to urine creatinine in mg/g). B. Box-whisker and scatter plots of urine total protein distributions in urine. (Top panels: urine total protein concentrations in mg/dL; Bottom panels: ratio of urine total protein to urine creatinine in mg/mg). Urine albumin, creatinine, and total protein were measured in urines obtained from 103 healthy donor by bead-based immunoassay, ELISA, and Bradford method, respectively. Boxes represent 25–75^th^ percentiles, whiskers represent range.

Urines in Set I were found to contain albumin at a mean level of 0.74 mg/dL (95% CI: 0.46–1.01 mg/dL) and total protein at a mean level of 16.94 mg/dL (95% CI: 14.32–19.56 mg/dL) (Figure 1A and 1B, respectively). Ratios of urine abumin to urine creatinine (ACR) and urine total protein to urine creatinine (PCR) are commonly used indicators of albuminuria and proteinuria, respectively, in spot-collected urines. In Set I the mean ACR was 4.56 mg/g (95% CI: 2.19–6.92 mg/g) while the mean PCR was 0.11 mg/mg (95% CI: 0.08–0.13 mg/mg) (Figure 1A and 1B, respectively). These values are within the nominal range for individuals with normal renal function and without chronic kidney disease.

### Relative Abundance of Urine Proteins

The mean and median levels for each of the 211 proteins evaluated in Set 1, along with coefficients of variation are listed in Table S2 in File S1. Absolute biomarker measurements and UCr-normalized values are presented in order of decreasing absolute observations. Of the 202 urine proteins measured using standard units, 62 were observed at levels of 1 ng/ml or greater. The 62 proteins were divided into three classes, defined by relative concentrations, and are presented in Table 2. Class 1 consists of 8 low molecular weight proteins observed at levels ranging from 100 ng/ml to >10 ug/ml. THP demonstrated the greatest abundance of all evaluated proteins with other glycoproteins and high abundance plasma proteins predominating in class 1. Class 2 consists of 20 proteins ranging in concentration from 10–100 ng/ml. This class of proteins was considerably more diverse, including proteases, growth factors, immunological factors, soluble receptors, hormones and other protein types. Several members of class 2 (MPO, thrombospondin, MDA-LDL, α-2-macroglobulin), listed in bold, exhibit molecular weights exceeding 70 kDa. Class 3 consists of 34 proteins with concentrations ranging from 1–10 ng/ml. Like class 2, class 3 demonstrated considerable diversity in protein types and included several MMPs, cytokines, structural proteins and ECM components in addition to many of the types present in class 2. A number of high molecular weight proteins were present in class 3 including fibronectin, complement C3, CEA, complement H, apolipoprotein B, sgp130, sVCAM-1 and NCAM. The five most abundant urine proteins utilizing non-standard units, classified as Class 4, are also included in Table 2. Included among these proteins were several pituitary hormones (FSH, LH) and several mucin-associated glycoprotein carbohydrate antigens (CA 15-3, CA 125, CA 19-9). Surprisingly, the normalization of biomarker measurements based on UCr levels did not appreciably alter the relative abundance of the proteins. When absolute biomarker measurements were compared directly against UCr-normalized values for each analyte the two were observed to correlate to a high degree (r^2^ = .9997, p<.0001).

**Table 2:** Relative Abundance classes of urine protein biomarkers.

Class 1: 100 ng/ml–>10 ug/ml		Class 2: 10 ng/ml –100 ng/ml		Class 3: 1 ng/ml –10 ng/ml		Class 4: Non-standard units	
Biomarker	Protein Family	Biomarker	Protein Family	Biomarker	Protein Family	Biomarker	Protein Family
THP	glycoprotein	IGFBP-7	growth modulator	Transthyretin	carrier protein	FSH	hormone
HSA	plasma Protein	HE4	glycoprotein	Kallikrein 10	serine protease	LH	hormone
SCC	glycoprotein	PSA	serine protease	Apolipoprotein AII	apolipoprotein	CA 15-3	cancer antigen
OPN	cytokine	EGF	growth factor	Apolipoprotein E	apolipoprotein	CA-125	cancer antigen
Calbindin	calcium binding	**MPO**	peroxidase	NSE	enolase	CA 19-9	cancer antigen
Clusterin	glycoprotein	Cystatin C	Protease Inhibitor	M-CSF	cytokine		
Mammaglobin	glycoprotein	β2-Microglobulin	MHC-1 component	TIMP-2	MMP inhibitor		
α1-Antitrypsin	protease Inhibitor	IGFBP-3	growth modulator	**Fibronectin**	extracellular matrix		
		fPSA	protease	ANGPTL4	glycoprotein		
		**Thrombospondin**	Anti-angiogenesis	GSTα	enzyme		
		LOX-1	membrane receptor	sTNFRII	secreted receptor		
		GST π	enzyme	MMP-8	MMP		
		TFF-3	gut secretion	**Complement C3**	complement		
		C-Peptide	peptide	**CEA**	glycoprotein		
		Thrombomodulin	receptor	sICAM-1	adhesion molecule		
		**MDA-LDL**	lipoprotein	Keratin-1,10,11	structural protein		
		Adiponectin	hormone	Cortisol	hormone (steroid)		
		Cathepsin D	protease	Angiogenin	ribonuclease		
		Apolipoprotein A1	apolipoprotein	**Complement H**	complement		
		**α-2-Macroglobulin**	plasma Protein	Involucrin	envelope protein		
				**Apolipoprotein B**	apolipoprotein		
				**sgp130**	cytokine		
				MMP-7	MMP		
				MMP-9	MMP		
				H-FABP	growth inhibitor		
				**sVCAM-1**	adhesion molecule		
				PBEF	enzyme		
				Complement C4	complement		
				Keratin-6	structural protein		
				sTNFRI	secreted receptor		
				Cytokeratin 19	intermediate filament		
				Endostatin	extracellular signalling		
				OC	hormone		
				**NCAM**	adhesion molecule		


Biomarkers within each class listed in order of decreasing relative abundance; Biomarkers in bold indicate high molecular weight (>70 kDa).

### Population variability of urine biomarkers

**Figure 2:**
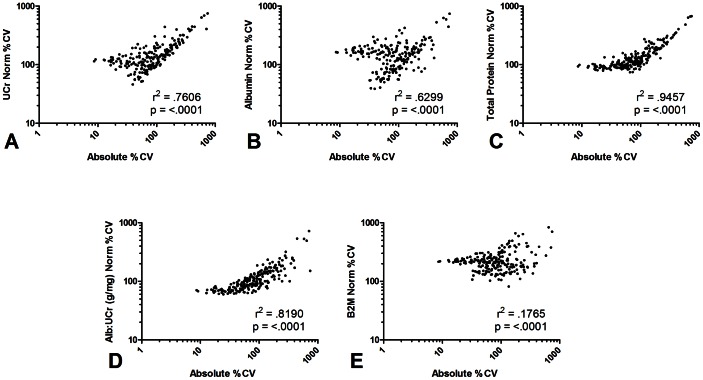
Effect of several normalization methods on the population variability of urine proteins. Urines obtained from 103 healthy donors were evaluated for levels of 211 proteins using multiplexed bead-based immunoassays. Coefficients of variation (CV) were determined for each of the 211 urine proteins based on absolute and normalized values. Correlation between the two sets of values was evaluated using Pearson's test of correlation. Normalized values were calculated by dividing absolute biomarker concentration by the level of several urine parameters: **A**. urine creatinine (UCr); **B**. urine albumin; **C**. urine total protein; **D**. ratio of urine albumin to urine creatinin (ACR); **E**. β-2-microglobulin.

CVs were calculated for each evaluated analyte in an effort to gauge the population variability associated with its presence in urine. Observed CVs for absolute measurements and UCr normalized data are presented in Table S2 in File S1 and ranged from greater than 700% to less than 10%. The impact of several methods of normalization on biomarker variability was assessed using the Pearson test of correlation (Figure 2). In addition to UCr, biomarker measurements were normalized based on urine albumin, a marker of glomerular integrity, and β-2-macroglobulin (B2M), a marker of tubular secretion. Normalization based on total urine protein content and the albumin to creatinine ratio (ACR) was also evaluated. In each case, absolute and normalized CVs correlated significantly (p<.0001), however r^2^ values varied considerably. Normalization based on total protein (Figure 2C) produced the smallest impact on biomarker variability, according to the r^2^ value, while normalization based on B2M (Figure 2E) had the most profound effect. Normalization based on UCr, albumin, and ACR (Figure 2A,B,D) had the greatest impact on low-variability biomarkers. A general trend was observed wherein biomarkers exhibiting low absolute CVs demonstrated progressively higher CVs following normalization to ACR, UCr, and albumin, respectively. It was further observed that proteins demonstrating the highest levels of absolute variability did so independent of normalization by any method. This was particularly true for proteins with CVs greater than 400% including: CA 19-9, serum amyloid A (SAA), heart type-fatty acid binding protein (H-FABP), myeloperoxidase (MPO), squamous cell carcinoma antigen (SCC) and apolipoprotein AII.

### Urine biomarkers correlated with urine creatinine (UCr), albumin, and beta-2-microglobulin (B2M)

Each of the biomarkers tested in Set I was evaluated for correlation with UCr, albumin, and B2M in order to assess whether each biomarker might be associated with glomerular filtration rate, glomerular integrity, and tubular secretion, respectively. Biomarkers identified to be significantly and uniquely correlated to each urine parameter are listed in Table 3. Many additional biomarkers were observed to be significantly correlated to multiple parameters (data not shown). Both positive and negative correlations were observed in relation to UCr, while all and nearly all correlations to albumin and B2M, respectively were in the postitive direction. The strongest correlations, in terms of r coefficient, were observed in relation to albumin. Many of the biomarkers identified in this analysis were listed among classes 2 and 3 in the relative abundance analysis above. Among the class 1 biomarkers, SCC and alpha-1-antitrypsin were correlated to albumin while clusterin and THP were correlated to B2M.

**Table 3:** Urine biomarkers correlating to specific urine parameters.

Urine Creatinine		Urine Albumin		Urine B2M	
Biomarker	Pearson r	Biomarker	Pearson r	Biomarker	Pearson r
Amphiregulin	0.539	Apo AII	0.901	sIL-1RII	0.767
LOX-1	0.515	CA-125	0.889	Prolactin	0.754
sE-Selectin	−0.414	Angiostatin	0.881	Adiponectin	0.715
EGF	0.407	Ang-2	0.872	CA 15-3	0.687
CCL19/MIP3β	−0.390	TFF-3	0.858	Flt-3L	0.501
Thrombospondin	0.381	CEA	0.834	GST π	0.452
sVEGFR2	0.370	Complement C4	0.832	PP	0.447
TIMP-3	0.357	Keratin-110-11	0.831	Fractalkine	0.440
Renin	−0.338	a-2-Macroglobulin	0.822	IL-11	0.430
IL-21	−0.337	Eotaxin-2	0.819	GSTα	0.398
GLP-1 (active)	−0.325	BCA-1	0.810	ANGPTL6/AGF	0.395
MMP-3	0.322	TARC	0.769	sIL-1RI	0.395
Ghrelin (active)	−0.319	CXCL11/I-TAC	0.743	TRAIL	0.342
HSP 70	−0.318	Apo A1	0.736	Complement C3	0.337
FGF-23	−0.306	BLC/CXCL13	0.688	Betacellulin	0.317
PIGF	0.300	HSP27(Total)	0.671	Clusterin	0.317
TIMP-4	0.297	MMP-2	0.649	Tenascin C	0.298
MMP-12	−0.291	CXCL9/MIG	0.632	sVEGFR2	0.298
IL-1b	−0.289	Perforin	0.624	TIMP-1	0.295
LPS	0.277	IGF-1R	0.565	GLP-1 (active)	0.292
sIL-4R	−0.272	Complement H	0.537	CA72-4	0.287
PSA	0.257	CD-105	0.524	CXCL9/MIG	0.263
FABP1	−0.250	Apo B	0.507	OPG	0.260
6CKine	−0.250	CXCL6/GCP2	0.495	IGFBP-1	0.253
CTACK	−0.246	SCC	0.478	Leptin	0.249
fPSA	0.227	pHSP27	0.476	TSH	0.246
IL-33	−0.222	CCL20/MIP3α	0.458	NT-Pro-BNP	−0.241
		IL-8	0.450	THP	0.227
		MMP-8	0.439		
		BDNF	0.419		
		Kallikrein 10	0.391		
		MMP-9	0.383		
		a1-Antitrypsin	0.371		
		Granzyme A	0.368		
		CA 15-3	0.307		
		Involucrin	0.300		
		AGRP	0.284		
		CA72-4	0.277		
		HSP60	0.259		

All correlations significant with p<0.05 by the Pearson test of correlation.

### Western blot analysis of selected urine proteins

**Figure 3:**
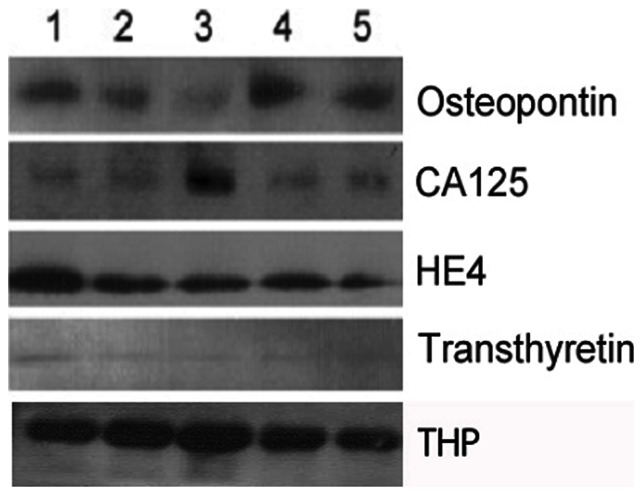
Western blot of OPN, CA 125, HE4, and TTR from human urine. A representative western blot of Osteopontin (OPN), CA125, HE4, and transthyretin (TTR) antigens expressed in protein lysates obtained from concentrated human urine samples from healthy individuals is depicted. Anti-Osteopontin, HE4, and TTR antibodies detected a single protein isoform of the corresponding proteins. Osteopontin migrated with an apparent molecular mass of 60 kDa, HE4 protein fragment migrated at 13 kDa, and TTR at 15 kDa. Anti-CA125 antibody detected three different protein fragments. The major CA125 fragment migrated in SDS-PAGE with an apparent molecular mass of 41 kDa (shown) and two smaller protein bands were in the range of ∼ 28–30 kDa. The presence of the different CA125 protein fragments in the human urine samples was confirmed by immunoblotting using various anti-CA125 antibodies. THP (68 kDa band) was evaluated as a loading control.

In order to provide confirmation of the multiplexed immunoassay results, several urine proteins were evaluated by western blot. The selected proteins are of particular interest to cancer biomarker research and were observed at relatively high abundance by immunoassay. A representative western blot for osteopontin (OPN), CA 125, HE4, and transthyretin (TTR) is presented in Figure 3. Monoclonal antibodies directed against osteopontin, HE4, and TTR detected a single protein isoforms of each. Osteopontin migrated with an apparent molecular mass of 60 kDa, while HE4 and TTR migrated at 13 and 15 kDa, respectively. A monoclonal antibody directed against CA 125 detected three distinct protein fragments. The major CA125 fragment migrated in SDS-PAGE with an apparent molecular mass of 41 kDa (shown) and two smaller protein bands were in the range of ∼28–30 kDa. The presence of each CA 125 protein fragments detected in human urine was confirmed using several additional anti-CA 125 antibodies (data not shown). A highly abundant 68kDa fragment corresponding to THP was visualized in each sample.

### Temporal Variation in Urine Biomarker Measurements

**Figure 4:**
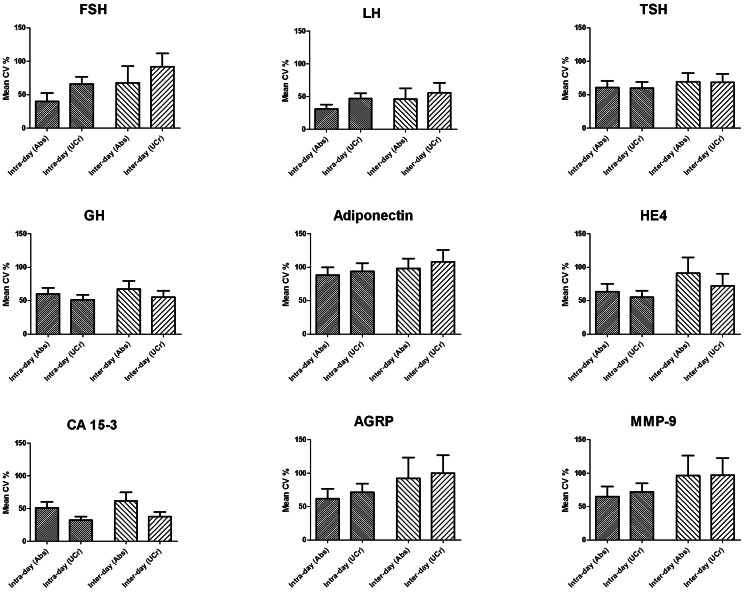
Temporal variability of urine biomarkers. Urines were collected three times a day (day, evening, night) over a two day period from 25 healthy female donors. Each urine sample was evaluated for 29 biomarkers by multiplexed immunoassay. Coefficients of variation (CV) were calculated for each biomarker among the three samples obtained each day (intra-day) and among the six samples obtained over the two day period (inter-day). Mean CVs for each reproducibly detectable biomarker in the entire 25 donor set are presented. Abs – absolute measurements; UCr – measurements normalized to levels of urine creatinine. Error bars represent 95% confidence intervals.

Among the 29 biomarkers evaluated in the Set II urines, 9 were deemed evaluable based on reproducible measurements greater than two standard deviations above the blank used for each immunoassay. For each donor, CVs were determined among the three measurements (day, evening, night) obtained on each day of collection, and the for the six total measurements obtained over the course of two days. CVs were calculated separately for absolute biomarker measurements and UCr-normalized measurements. The average CVs (with 95% confidence interval) for each biomarker in the entire group of 25 donors are presented in Figure 4. Among the nine evaluated biomarkers, consistently low variability was observed for LH, TSH, and GH, while adiponectin displayed a persistently high level of variability. Each of the other biomarkers displayed levels of variability which fluctuated considerably based on intra- versus inter-day measurements or UCr normalization. As expected inter-day variability was increased to some extent over intra-day variability in all nine biomarkers, and most notably in FSH, HE4, AGRP, and MMP-9. UCr normalization resulted in a marked increase in variability in FSH and marked decreased in variability in GH, HE4, and CA 15-3.

## Discussion

Our analysis of protein biomarkers in the urine of healthy donors revealed the presence of over 200 distinct proteins present at varying levels of abundance and population variability. The potential applications of the measurement of these biomarkers in healthy and diseased individuals are far reaching. An initial assessment of the renal health of the individuals was included in our study in the absence of available serum creatinine values as the early stages of kidney disease are often asymptomatic and the presence of albuminuria or proteinuria would serve as a significant confounding factor. The observed ACR and PCR for Set I individuals fell within normal levels, 10 mg/g and 0.15 mg/mg, respectively [20], [21]. Here we report on the informative potential of protein biomarkers present within the low physiological range of urine protein content.

The observed concentration classes presented in Table 2 were defined by the investigators in order to illustrate several trends present within our findings. The high abundance class 1 biomarkers included low molecular weight proteins likely to pass freely through the glomerular filtration complex or be secreted by cells of the renal tubule. As expected, THP and HSA were the two most abundant urine proteins evaluated. THP, a glycophosphoinositol-anchored glycoprotein shed into the urine by cells in the thick ascending loop of Henle, is known to be the most abundant protein found in normal urine. HSA is the most abundant protein in blood plasma and it is estimated that approximately 0.06% of plasma albumin is transferred into urine through glomerular filtration [22]. Several other members of class 1 have been previously examined as biomarkers of renal and urological health including calbindin [23], clusterin [24]–[26], A1A [27]–[29], and MPO [30]. Urine levels of OPN, a multifunctional protein implicated in bone remodeling and inflammation, have been associated with rheumatoid arthritis and ovarian cancer [4], [31]. The list of biomarkers included in classes 2 and 3 illustrate the diverse potential of urine biomarkers and include proteins involved in inflammation, apoptosis, growth regulation, metabolism, endocrine signaling and numerous additional processes. These classes also include several proteins with molecular weights exceeding the predicted size limit for glomerular filtration, reported as 40–60 kDa [32]. Our findings are consistent with two recent reports describing the analysis of glycoproteins in urine from bladder cancer patients and healthy controls in which a diverse array of protein biomarkers demonstrating considerable variability in size and function were identified [33], [34]. Glycoproteins were enriched among the three classes of proteins presented here, and those previous reports demonstrated the potential utility of urine glycoproteins in cancer diagnostics. Among the high molecular weight proteins are the heavily glycosylated tumor markers CA 15-3, CA 125, and CA 19-9. The results of our western blot analysis suggest that proteolytic processing of large biomarkers may play a role in their secretion into urine. Alternatively, large molecular weight proteins may reach the urine via exosomes, a process which has been described by others [35].

Several urine biomarkers relevant to cancer research including HE4, OPN, CA 125 and TTR were examined using WB in order to confirm their presence in urine (Figure 3). HE4 is a secreted glycoprotein which was recently identified in the urine of patients with serous ovarian carcinoma [36]. Our analysis of healthy individuals identified a major HE4 isoform of 13 kDa. This is consistent with a previous report in which five isoforms of HE4 were identified ranging from 8–13 kDa [37]. Our WB analysis indicated the presence of a single 60 kDa OPN fragment with slightly variable expression among healthy volunteers. This is consistent with previous studies in which urinary OPN was identified within a range of 55–66 kDa [38], [39]. CA125, a mucin-associated glycoprotein highly overexpressed in ovarian carcinomas, has been detected in serum, urine and saliva of patients with adnexal masses and at elevated levels in the urine of patients with bladder carcinoma [40], [41]. Fully glycosylated CA 125 exhibits a molecular weight of 1–5 MDa and is not expected to pass freely through glomerular capillaries. Thus, proteolytic processing is likely to factor in the observed urinary levels of CA 125 in this and prior studies. Our WB analysis revealed three CA 125 protein fragments with the most predominant band migrating at 41 kDa. A CA 125 protein fragment of similar size (40 kDa) was reported in the urine of patients with nephrotic syndrome [42]. TTR, a protein responsible for the transport of thyroxine from the bloodstream to the brain, is significantly elevated in the serum of patients with glioblastoma [43] and lung cancer [44]. TTR expression has also been detected in the urine of diabetes patients [45]. Here we show that urine from healthy donors contains a 15 kDa TTR isoform. This was comparable to the TTR isoform found in sera of lung cancer patients and in CSF of patients with first-onset psychosis [46], [47].

We speculate that three general components form the basis for urine biomarker variability in our healthy population: (1) variations in local and systemic tissue production of specific proteins; (2) mechanisms of renal elimination, including diurnal and genetic variation in renal physiology; and (3) variations in fluid intake resulting in the dilution or concentration of urine proteins. Normalization of urine biomarker measurements based on well characterized urine markers is intended to reduce the effect of the latter two components of variation in order to more precisely evaluate the biological mechanisms underlying the first. The comprehensive understanding of biological variability in healthy and diseased subjects forms the basis of the clinical utility of biomarkers. Our approach to the question of normalization was designed to consider variation in fluid intake (UCr), variation in glomerular integrity (albumin, ACR), and the tubular contributions to urine protein (B2M). We consider normalization based on urine total protein to be a holistic approach. The use of UCr as a normalizing factor did not lead to an overall reduction or enhancement of biomarker variability but did result in a moderate increase in variability among markers demonstrating absolute CV values below 100% (Figure 2). This observation was augmented when albumin was used as a normalization factor and less prevalent when ACR or total protein was used. Normalization based on B2M resulted in an elevation in variability for the majority of biomarkers under investigation. Our findings do not permit us to make any definitive conclusions on the most appropriate means of biomarker normalization. It is important to note that normalization factors such as UCr have been chosen based on demonstrated intra-subject stability, while their utility in population based applications remains in question. Our results do suggest that biomarkers demonstrating relatively high levels of absolute variability respond the least to the various methods of normalization. Further study may demonstrate that biomarkers of this type represent promising candidates for clinical use.

The use of serially collected urine permitted an estimate of intra- and inter-day variability in urine biomarker concentrations. The observed level of variability was significant with CVs reaching or exceeding 50% for the majority of evaluable proteins. As variability was determined on a intrapersonal basis over a short period of time, physiological variations regarding renal function and biomarker release were not expected to play a major role in this analysis, leaving variation in fluid intake as the major variability component. Thus, the observation that normalization based on UCr did not result in a marked reduction in variability in six of the nine evaluated proteins was unexpected. Furthermore, aside from a generally increased level of variability over the two-day span in comparison to intra-day measurements, no consistent pattern of variability among the biomarkers could be discerned. These results suggest that even on an intrapersonal level, variability in urine biomarker production is based on mechanisms specific to each protein.

The analysis of biomarker correlations revealed several broad arrays of proteins significantly correlated with UCr, albumin, B2M or a combination of the three. Individual proteins were observed to be both positively and negatively correlated to UCr. These observations, in combination with the relative abundance, variability, and temporal analyses detailed above, suggest to us that protein specific mechanisms of renal filtration play a significant role in the composition of the systemic component of the urinary proteome. At the level of glomerular filtration, a mechanistic explanation for our observations may be provided by a closer examination of the glomerular glycocalyx, a relatively thick layer of highly glycosylated, negatively charged proteins covering the glomerular endothelial fenestrae [48], [49]. The glycocalyx is composed of both cell surface-anchored proteins such as proteoglycans and sialoproteins as well as adsorbed components from plasma including albumin, orosomucoid and lumican [50]. Accumulating evidence indicates that the glycocalyx contributes greatly to the permeability of negatively charged proteins in the glomerulus (reviewed in [32], [51]). Based on our findings, we propose that protein-protein interactions between plasma biomarkers and the glomerular glycocalyx are critical in determining the filtered component of urine total protein. Such specificity in renal elimination would further complicate the selection of appropriate normalization methods and support the advancement of biomarkers demonstrating performance independent of normalization.

Several limitations characterize the current investigation, the most prominent of which is the inclusion of only healthy donors in our study population. The exclusion of diseased individuals was designed to enable a careful evaluation of renal physiology with regard to protein biomarker processing, however this does prevent any direct extension of our findings into specific clinical settings. The use of spot collected urines may also present a limitation in that it may serve to complicate matters of biomarker variability by not explicitly controlling for diurnal variation or fluid intake. However, this approach is likely to more closely approximate intended clinical applications wherein cost and logistic considerations may preclude timed collections. It should also be noted that our collection procedure did not include any manipulation of urine sample between collection and freezing in order to best preserve the protein content. As such, the cellular contribution of proteins derived from bladder or renal tissues or squamous epithelial cells from urethra or external genitalia cannot be ruled out.

In summary, an extensive and diverse analysis of protein biomarkers in urine yielded a profile of immunodetectable factors with a wide range of abundance and population variability. Several of these observations were confirmed by western blot. A correlation analysis between protein biomarkers and several urine parameters revealed a complex pattern of biomarker observations suggesting protein-specific mechanisms of glomerular filtration. The usefulness of several methods of normalization of urine biomarker measurements in the current study was informative but inconclusive. These findings should further enhance our understanding of urine proteomics and provide a basis for additional, more narrowly targeted analyses of clinically useful urinary protein biomarkers.

## Supporting Information

File S1 (DOCX)
