# Supplementary material for: An Extensive Targeted Proteomic Analysis of Disease-Related Protein Biomarkers in Urine from Healthy Donors
Source: PLoS One. 2013 May 28;8(5):e63368. doi: 10.1371/journal.pone.0063368 (PMC3665773; doi:10.1371/journal.pone.0063368)
Supplement: File S1 — (DOCX) [file pone.0063368.s001.docx]

**An extensive targeted proteomic analysis of disease-related protein biomarkers in urine from healthy donors**

Brian M. Nolen, Lidiya S. Orlichenko, Adele Marrangoni, Liudmila Velikokhatnaya, Denise Prosser,William Grizzle, Kevin Ho, Frank J. Jenkins, Dana H. Bovbjerg, and Anna E. Lokshin

**Supporting Information File S1**

| **Table S1. Complete list of biomarkers** | | |
| --- | --- | --- |
| **Biomaker** | **Full name/Alternative Name** | **Protein Type /Family** |
| 6CKine | CCL21 | Chemokine |
| α1-Antitrypsin | NA (Not applicable) | Protease Inhibitor |
| α-2-Macroglobulin | NA | Plasma Protein |
| ACTH | Adrenocorticotropic hormome | Hormone |
| Adiponectin | NA | Hormone |
| AFP | alpha-fetoprotein | glycoprotein |
| AGRP | agouti-related peptide | neuropeptide |
| Amphiregulin | NA | growth factor |
| Amylin | NA | Hormone (peptide) |
| Angiogenin | NA | Ribonuclease |
| Angiopoietin-2 | NA | growth factor |
| Angiostatin | NA | angiogenesis inhibitor |
| ANGPTL3 | angiopoietin-like protein 3 | glycoprotein |
| ANGPTL4 | angiopoietin-like protein 4 | glycoprotein |
| ANGPTL6/AGF | angiopoietin-like protien 5 | glycoprotein |
| Apolipoprotein A1 | NA | apolipoprotein |
| Apolipoprotein AII | NA | apolipoprotein |
| Apolipoprotein B | NA | apolipoprotein |
| Apolipoprotein Clll | NA | apolipoprotein |
| Apolipoprotein E | NA | apolipoprotein |
| β2-Microglobulin | NA | MHC-1 component |
| BCA-1/CXCL13 | B-cell attracting chemokine 1/CXCL13 | Chemokine |
| Bcl-2 | B-cell CCL/lymphoma 2 | Mitochondrial membrane |
| BDNF | brain derived neurotrophic factor | neurotrophin |
| Betacellulin | NA | glycoprotein |
| CA 15-3 | NA | Cancer antigen |
| CA 19-9 | NA | Cancer antigen |
| CA-125 | NA | Cancer antigen |
| CA72-4 | NA | Cancer antigen |
| Calbindin | NA | calcium binding protein |
| Cathepsin D | NA | Protease |
| CCL14a/HCC-1 | hemofiltrate CC chemokine 1 | Chemokine |
| CCL19/MIP3β | macrophage inflammatory protein 3-beta | Chemokine |
| CCL20/MIP3α | macrophage inflammatory protein 3-alpha | Chemokine |
| CD-105 | endoglin | TGFR family receptor |
| CEA | carcinoembryonic antigen | glycoprotein |
| Clusterin | NA | glycoprotein |
| CNTF | ciliary neurotrophic factor | cytokine |
| Complement H | NA | complement |
| Complement C3 | NA | complement |
| Complement C4 | NA | complement |
| Cortisol | NA | Hormone (steroid) |
| C-Peptide | NA | peptide |
| CRP | C-reactive protein | acute phase reactant |
| CTACK | cutaneous T-cell attracting chemokine/CCL27 | Chemokine |
| CXCL11/I-TAC | interferon-inducible T-cell alpha chemoattractant | Chemokine |
| CXCL6/GCP2 | granulocyte chemotactant protein | Chemokine |
| CXCL7/NAP2 | neutrophil activating peptide 2 | Chemokine |
| CXCL9/MIG | monocyte induced by gamma interferon | Chemokine |
| Cystatin C | NA | Protease Inhibitor |
| Cytokeratin 19 | NA | Intermediate filament |
| EGF | epidermal growth factor | growth factor |
| EGFR | epidermal growth factor receptor | growth factor receptor |
| ENA-78 | epithelial derived neutrophile activating protein 78/CXCL5 | Chemokine |
| Endostatin | NA | extracellular signalling |
| Eotaxin-2 | NA | Chemokine |
| Eotaxin-3 | NA | Chemokine |
| EPCAM | epithelial cell adhesion molecule | glycoprotein |
| Epiregulin | NA | growth factor |
| ErbB2 | human epidermal growth factor receptor 2 | growth factor receptor 2 |
| FABP1 | fatty acid binding protein 1 | carrier protein |
| FGF-19 | fibroblast growth factor 19 | growth factor |
| FGF-21 | fibroblast growth factor 21 | growth factor |
| FGF-23 | fibroblast growth factor 23 | growth factor |
| FGF-b | fibroblast growth factor beta | growth factor |
| Fibronectin | NA | extracellular matrix protein |
| Flt-3 Ligand | fms-related tyrosine kinase 3 ligand | cytokine |
| fPSA( | free prostate specific antigen | protease |
| Fractalkine | NA | cytokine |
| FSH | follicle stimulating hormone | hormone |
| GH | growth hormone | hormone |
| Ghrelin | NA | Hormone (peptide) |
| GIP | gastric inhibitory peptide | Hormone (peptide) |
| GLP-1 | glucagon-like peptide 1 | Hormone (peptide) |
| Glucagon | NA | Hormone (peptide) |
| Granzyme A | NA | Serine protease |
| Granzyme B | NA | Serine protease |
| GST π | glutathione S-transferase | enzyme |
| GSTα | glutathione S-transferase | enzyme |
| HE4 | human epididymal protein 4 | glycoprotein |
| H-FABP | heart type-fatty acide binding protein | growth inhibitor |
| HGF | hepatocyte growth factor | growth factor |
| HSA | human serum albumin | Plasma Protein |
| HSP27 | heat shock protein 27 | chaperone |
| HSP60 | heat shock protein 60 | chaperone |
| HSP70 | heat shock protein 70 | chaperone |
| HSP90a | heat shock protein 90a | chaperone |
| I-309 | NA | Chemokine |
| IGF-1R | insulin-like growth factor 1 receptor | receptor |
| IGFBP-1 | insulin-like growth factor binding protein 1 | growth modulator |
| IGFBP-2 | insulin-like growth factor binding protein 2 | growth modulator |
| IGFBP-3 | insulin-like growth factor binding protein 3 | growth modulator |
| IGFBP-4 | insulin-like growth factor binding protein 4 | growth modulator |
| IGFBP-5 | insulin-like growth factor binding protein 5 | growth modulator |
| IGFBP-6 | insulin-like growth factor binding protein 6 | growth modulator |
| IGFBP-7 | insulin-like growth factor binding protein 7 | growth modulator |
| IL-11 | interleukin 11 | cytokine |
| IL-16 | interleukin 16 | cytokine |
| IL-1b | interleukin 1 beta | cytokine |
| IL-20 | interleukin 20 | cytokine |
| IL-21 | interleukin 21 | cytokine |
| IL-23 | interleukin 23 | cytokine |
| IL-28A | interleukin 28 alpha | cytokine |
| IL-29 | interleukin 29 | cytokine |
| IL-33 | interleukin 33 | cytokine |
| IL-6 | interleukin 6 | cytokine |
| IL-8 | interleukin 8 | cytokine |
| INF-b | interferon beta | cytokine |
| INF-ω | interferon omega | cytokine |
| Insulin | NA | hormone |
| Involucrin | NA | envolope protein |
| Kallikrein 10 | NA | Serine protease |
| Keratin-1,10,11 | NA | structural protein |
| Keratin-6 | NA | structural protein |
| KIM-1 | kidney injury molecule | transmembrane protein |
| Leptin | NA | extracellular signalling |
| LH | luteinizing hormone | hormone |
| LIF | leukemia inhibitory factor | cytokine |
| LOX-1 | lectin type oxidized LDL receptor 1 | transmembrane receptor |
| LPS | lipopolysaccharide | carbohydrate antigen |
| Mammaglobin | NA | glycoprotein |
| MCP-1 | monocyte chemotactic protein 1/CCL2 | chemokine |
| MCP-2 | monocyte chemotactic protein 2/CCL8 | chemokine |
| MCP-4 | monocyte chemotactic protein 2/CCL13 | chemokine |
| M-CSF | macrophage colony stimulating factor | cytokine |
| MDA-LDL | malondialdehyde modified low density lipoprotein | lipoprotein |
| MICA | MHC class 1 chain related protein A | immune recognition |
| MIF | macrophage migration inhibitory factor | cytokine |
| MIP-1d | macrophage inflammatory protein 1 delta | Chemokine |
| MIP-4 | macrophage inflammaotry protein 4 | Chemokine |
| MMP-1 | matrix metalloproteinase 1 | MMP |
| MMP-12 | matrix metalloproteinase 12 | MMP |
| MMP-13 | matrix metalloproteinase 13 | MMP |
| MMP-2 | matrix metalloproteinase 2 | MMP |
| MMP-3 | matrix metalloproteinase 3 | MMP |
| MMP-7 | matrix metalloproteinase 7 | MMP |
| MMP-8 | matrix metalloproteinase 8 | MMP |
| MMP-9 | matrix metalloproteinase 9 | MMP |
| MPO | myeloperoxidase | endogenous peroxidase |
| NCAM | neural cell adhesion molecule | adhesion molecule |
| NGF | neural growth factor | growth factor |
| NSE | neuron specific enolase | enolase |
| NT-Pro-BNP | N-terminal prohormone of brain natriuretic peptide | Hormone (peptide) |
| OC | osteocalcin | hormone |
| Oncostatin | NA | cytokine |
| OPG | osteoprotegerin | cytokine receptor |
| OPN | osteopontin | cytokine |
| PAI-1 | plasminogen activator inhibitor 1 | serpin |
| PBEF | pre-B cell enhancing factor/visfatin | enzyme |
| PDGF-AA | platelet derived growth factor AA | growth factor |
| PDGF-AB/BB | platelet derived growth factor AB/BB | growth factor |
| PDGF-BB | platelet derived growth factor BB | growth factor |
| PEDF | pigment epithelium derived factor | serpin |
| Perforin | NA | cytolytic factor |
| Phospho-HSP27 | heat shock protein 27 (phosphorylated) | chaperone |
| PIGF | placental growth factor | growth factor |
| PP | pancreatic polypeptide | hormone |
| Progesterone Receptor | NA | steroid receptor |
| Prolactin | NA | hormone |
| PSA | prostate specific antigen | Serine protease |
| PTH | parathyroid hormone | hormone |
| PYY | peptide YY | Hormone (peptide) |
| RANKL | Receptor activator of nuclear factor kappa-B ligand | cytokine |
| RANTES | regulated and normal T cell expressed and secreted/CCL5 | cytokine |
| Renin | NA | protease |
| SAA | serum amyloid A | acute phase reactant |
| SCC | squamous cell carcinoma antigen | glycoprotein |
| sCD137/4-1BB | NA | TNFR family receptor |
| sCD30 | NA | TNFR family receptor |
| sCD40L | NA | T-cell antigen |
| SCF | stem cell factor | cytokine |
| SDF-1a+B | stromal cell derived factor 1 (alpha and beta) | chemokine |
| sEGFR | soluble epidermal growth factor receptor | secreted receptor |
| sE-Selectin | NA | secreted adhesion molecule |
| sFas | NA | secreted receptor |
| sFasL | NA | apoptosis ligand |
| sgp130 | NA | cytokine |
| sICAM-1 | soluble intercellular adhesion molecule 1 | secreted adhesion molecule |
| sIL-1RI | soluble interleukin 1 receptor 1 | secreted receptor |
| sIL-1RII | soluble interleukin 1 receptor 2 | secreted receptor |
| sIL-2Rα | soluble interleukin 2 receptor alpha | secreted receptor |
| sIL-4R | soluble interleukin 4 receptor | secreted receptor |
| sIL-6R | soluble interleukin 6 receptor | secreted receptor |
| sRAGE | soluble receptor for advanced glycation endproducts | secreted receptor |
| sTNFRI | soluble tumor necrosis factor receptor 1 | secreted receptor |
| sTNFRII | soluble tumor necrosis factor receptor 2 | secreted receptor |
| sVCAM-1 | soluble vascular cell adhsion molecule 1 | secreted adhesion molecule |
| sVEGFR1 | soluble vascular endothelial growth factor receptor 1 | secreted receptor |
| sVEGFR2 | soluble vascular endothelial growth factor receptor 2 | secreted receptor |
| sVEGFR3 | soluble vascular endothelial growth factor receptor 3 | secreted receptor |
| TARC | thymus and activation related chemokine/CCL17 | chemokine |
| Tenascin C | NA | ECM protein |
| TFF-3 | trefoil factor 3 | Gut secretion |
| Tg II | tissue transglutaminase | enzyme |
| TGF-α | transforming growth factor alpha | growth factor |
| THP | tamm-horsfall protein | glycoprotein |
| Thrombomodulin | NA | receptor |
| Thrombospondin | NA | angiogenesis inhibitor |
| TIMP-1 | tissue inhibitor of metalloproteinases 1 | MMP inhibitor |
| TIMP-2 | tissue inhibitor of metalloproteinases 2 | MMP inhibitor |
| TIMP-3 | tissue inhibitor of metalloproteinases 3 | MMP inhibitor |
| TIMP-4 | tissue inhibitor of metalloproteinases 4 | MMP inhibitor |
| TNFα | tumor necrosis factor alpha | cytokine |
| tPAI-1 | tissue plasminogen activator inhibitor | serpin |
| TPO | thyroperoxidase | peroxidase |
| TRAIL | TNF-related apoptosis-inducing ligand | cytokine |
| TSH | thryoid stimulating hormone | hormone |
| Transthyretin | prealbumin | carrier protein |
| TSLP | thymic stromal lymphopoetin | cytokine |
| VEGF | vascular endothelial growth factor | growth factor |
| XCL1/Lymphotactin | NA | chemokine |

| **Table S2. Distributions of Urine Biomarkers in Healthy Individuals** | | | | | | | |
| --- | --- | --- | --- | --- | --- | --- | --- |
|  | **Absolute Concentrations (pg/ml)** | | |  | **Normalized Values†** | | |
| **Biomarker** | **Median** | **Mean** | **%CV** |  | **Median** | **Mean** | **%CV** |
| THP | 2356000 | 14930000 | 367.68 |  | 11256 | 94070 | 375.04 |
| HSA | 4678500 | 7359630 | 163.48 |  | 24730 | 45564 | 228.71 |
| SCC | 7506 | 686617 | 444.30 |  | 35.34 | 3330 | 442.02 |
| OPN | 669028 | 651139 | 49.85 |  | 2859 | 3077 | 56.02 |
| Calbindin | 103000 | 165461 | 104.71 |  | 445.0 | 722.2 | 105.47 |
| Clusterin | 61638 | 129116 | 253.12 |  | 334.4 | 773.9 | 317.16 |
| Mammaglobin | 93333 | 125928 | 106.08 |  | 434 | 680 | 123.00 |
| α1-Antitrypsin | 61272 | 115073 | 218.52 |  | 218.7 | 737.1 | 398.27 |
| IGFBP-7 | 81838 | 74392 | 62.70 |  | 264.8 | 341.9 | 72.80 |
| HE4 | 60754 | 71647 | 79.83 |  | 278.5 | 369.8 | 90.16 |
| PSA | 2532 | 68879 | 159.18 |  | 83.44 | 309.6 | 173.93 |
| EGF | 74183 | 66777 | 41.99 |  | 383.7 | 395.8 | 68.34 |
| MPO | 16082 | 64982 | 185.61 |  | 9.61 | 303.8 | 282.57 |
| Cystatin C | 49337 | 58673 | 87.42 |  | 170.3 | 265.7 | 105.45 |
| β2-Microglobulin | 36281 | 41833 | 105.02 |  | 139.5 | 203.2 | 131.67 |
| IGFBP-3 | 34863 | 31860 | 42.64 |  | 127.0 | 157.7 | 56.21 |
| fPSA | 105.0 | 30920 | 150.26 |  | 2.10 | 148.1 | 182.49 |
| Thrombospondin | 19559 | 28632 | 97.31 |  | 92.09 | 145.6 | 91.87 |
| LOX-1 | 20100 | 23734 | 83.38 |  | 87.75 | 99.70 | 97.54 |
| GST π | 6433 | 18006 | 144.67 |  | 34.69 | 86.64 | 149.30 |
| TFF-3 | 10873 | 16223 | 245.55 |  | 60.53 | 100.1 | 233.70 |
| C-Peptide | 14933 | 15090 | 42.76 |  | 74.44 | 90.80 | 74.51 |
| Thrombomodulin | 15141 | 14476 | 39.22 |  | 71.28 | 74.13 | 45.99 |
| MDA-LDL | 1550 | 13350 | 140.41 |  | 17.34 | 125.0 | 178.23 |
| Adiponectin | 9507 | 12836 | 99.76 |  | 48.47 | 73.04 | 98.65 |
| Cathepsin D | 12519 | 12791 | 51.52 |  | 62.73 | 74.35 | 73.34 |
| Apolipoprotein A1 | 4980 | 11824 | 138.39 |  | 33.39 | 67.51 | 120.89 |
| α-2-Macroglobulin | 2270 | 10649 | 243.56 |  | 13.44 | 61.03 | 240.12 |
| Transthyretin | 6240 | 9514 | 103.74 |  | 36.22 | 66.72 | 129.20 |
| Kallikrein 10 | 3644 | 8228 | 130.44 |  | 14.47 | 42.65 | 147.07 |
| Apolipoprotein AII | 1630 | 7870 | 403.96 |  | 7.65 | 39.65 | 446.99 |
| Apolipoprotein E | 5440 | 7548 | 134.97 |  | 28.48 | 42.47 | 142.84 |
| NSE | 1720 | 5596 | 122.08 |  | 14.57 | 30.47 | 149.15 |
| M-CSF | 5013 | 5212 | 55.00 |  | 21.70 | 24.35 | 51.60 |
| TIMP-2 | 4475 | 4604 | 67.91 |  | 15.02 | 21.66 | 80.50 |
| Fibronectin | 2238 | 4393 | 220.95 |  | 12.53 | 21.60 | 200.80 |
| ANGPTL4 | 3260 | 4078 | 64.30 |  | 15.74 | 32.47 | 157.89 |
| GSTα | 465 | 3974 | 284.33 |  | 2.52 | 20.60 | 237.84 |
| sTNFRII | 3710 | 3973 | 45.27 |  | 17.28 | 21.46 | 83.96 |
| MMP-8 | 873 | 3361 | 198.69 |  | 3.79 | 19.05 | 218.36 |
| Complement C3 | 1580 | 3295 | 348.28 |  | 6.16 | 18.09 | 407.95 |
| CEA | 1181 | 3265 | 270.19 |  | 7.77 | 24.04 | 267.79 |
| sICAM-1 | 2057 | 2900 | 86.87 |  | 11.34 | 15.60 | 95.16 |
| Keratin-1,10,11 | 2490 | 2851 | 87.32 |  | 11.83 | 21.10 | 125.35 |
| Cortisol | 2574 | 2795 | 37.04 |  | 14.39 | 25.83 | 151.52 |
| Angiogenin | 2502 | 2694 | 81.96 |  | 10.75 | 14.25 | 99.29 |
| Complement Factor H | 403.0 | 2571 | 223.90 |  | 2.68 | 11.13 | 194.14 |
| Involucrin | 2022 | 2450 | 92.65 |  | 11.34 | 14.27 | 94.20 |
| Apolipoprotein B | 1480 | 2435 | 187.13 |  | 7.09 | 17.23 | 157.42 |
| sgp130 | 2263 | 2415 | 48.59 |  | 11.33 | 12.76 | 61.02 |
| MMP-7 | 1181 | 2334 | 175.49 |  | 3.74 | 12.01 | 217.24 |
| MMP-9 | 500 | 2259 | 154.90 |  | 2.35 | 12.57 | 165.00 |
| H-FABP | 501.0 | 2248 | 646.08 |  | 2.49 | 15.46 | 687.04 |
| sVCAM-1 | 828 | 2086 | 123.08 |  | 4.90 | 11.43 | 145.53 |
| PBEF | 1616 | 1882 | 51.42 |  | 7.78 | 14.84 | 133.29 |
| Complement C4 | 345 | 1806 | 367.48 |  | 1.96 | 9.50 | 390.10 |
| Keratin-6 | 642.0 | 1693 | 216.53 |  | 3.18 | 9.84 | 198.93 |
| sTNFRI | 1347 | 1527 | 57.37 |  | 6.16 | 7.77 | 106.17 |
| Cytokeratin 19 | 860 | 1354 | 126.98 |  | 4.06 | 13.07 | 220.36 |
| Endostatin | 901.0 | 1296 | 140.17 |  | 3.85 | 6.51 | 135.33 |
| OC | 1162 | 1193 | 42.63 |  | 5.96 | 6.99 | 55.24 |
| NCAM | 1088 | 1062 | 48.89 |  | 5.43 | 5.99 | 61.95 |
| MIF | 466 | 787.3 | 106.70 |  | 2.09 | 3.53 | 115.18 |
| SAA | 54.90 | 776.8 | 709.35 |  | 0.301 | 3.01 | 406.17 |
| TGF-α | 658.0 | 729.4 | 82.01 |  | 3.36 | 4.22 | 87.29 |
| sIL-6R | 640.0 | 656.2 | 64.85 |  | 2.65 | 3.22 | 70.11 |
| CRP | 151.0 | 624.0 | 276.79 |  | 0.730 | 3.90 | 255.98 |
| PEDF | 233.0 | 534.4 | 210.74 |  | 1.04 | 2.98 | 228.99 |
| KIM-1 | 465.0 | 533.5 | 80.31 |  | 2.18 | 2.94 | 83.77 |
| TIMP-1 | 320.0 | 515.7 | 111.39 |  | 1.43 | 2.46 | 134.43 |
| MMP-2 | 364.0 | 453.9 | 104.40 |  | 1.57 | 2.31 | 126.19 |
| TIMP-3 | 451.0 | 422.8 | 68.21 |  | 1.60 | 2.45 | 132.62 |
| IGFBP-2 | 270.0 | 418.4 | 105.87 |  | 1.45 | 4.04 | 192.54 |
| IGFBP-5 | 317.0 | 405.0 | 182.64 |  | 1.30 | 3.44 | 182.28 |
| LPS | 21.50 | 400.4 | 169.34 |  | 0.174 | 1.64 | 169.40 |
| Apolipoprotein Clll | 326.0 | 396.1 | 97.66 |  | 1.48 | 2.05 | 94.99 |
| sEGFR | 356.0 | 395.2 | 57.18 |  | 1.87 | 2.12 | 72.83 |
| sVEGFR2 | 344.0 | 391.4 | 51.01 |  | 1.75 | 2.43 | 141.06 |
| PDGF-AA | 326.0 | 371.6 | 80.97 |  | 1.47 | 1.85 | 84.03 |
| TPO | 272.0 | 352.5 | 66.30 |  | 1.39 | 2.99 | 208.23 |
| sIL-2Rα | 324.0 | 340.1 | 68.70 |  | 1.39 | 1.70 | 75.52 |
| IGFBP-4 | 306.0 | 339.6 | 46.19 |  | 1.48 | 2.81 | 135.20 |
| FGF-23 | 314.0 | 331.1 | 21.83 |  | 1.66 | 2.64 | 125.77 |
| Angiostatin | 156.0 | 306.4 | 332.02 |  | 0.794 | 2.16 | 295.03 |
| EGFR | 268.0 | 272.8 | 31.35 |  | 1.53 | 1.85 | 88.70 |
| FGF-b | 272.0 | 267.1 | 20.26 |  | 1.60 | 1.91 | 82.40 |
| ANGPTL6/AGF | 252.0 | 249.2 | 12.72 |  | 1.29 | 1.91 | 121.15 |
| MCP-1 | 204.0 | 241.9 | 66.82 |  | 0.972 | 1.19 | 76.99 |
| HGF | 206.0 | 240.4 | 69.60 |  | 1.09 | 1.25 | 68.45 |
| Insulin | 188.0 | 233.6 | 60.65 |  | 1.13 | 2.21 | 194.79 |
| ANGPTL3 | 208.0 | 222.7 | 37.34 |  | 1.08 | 1.54 | 100.80 |
| IGFBP-1 | 87.40 | 203.36 | 165.58 |  | 0.530 | 1.18 | 145.59 |
| IL-23 | 121.0 | 192.2 | 91.22 |  | 0.635 | 1.84 | 203.79 |
| FABP1 | 148.0 | 189.3 | 52.48 |  | 0.867 | 1.68 | 187.38 |
| IGFBP-6 | 99.70 | 175.8 | 144.58 |  | 0.608 | 1.06 | 157.61 |
| HSP27 | 21.80 | 166.2 | 174.41 |  | 0.120 | 0.804 | 195.78 |
| Leptin | 143.0 | 156.4 | 63.10 |  | 0.727 | 0.985 | 118.93 |
| ErbB2 | 155.0 | 151.5 | 47.63 |  | 0.819 | 0.962 | 96.44 |
| HSP70 | 39.60 | 139.96 | 190.73 |  | 0.254 | 0.604 | 155.87 |
| Angiopoietin-2 | 114.0 | 116.5 | 59.33 |  | 0.517 | 0.746 | 109.09 |
| Renin | 60.40 | 115.5 | 114.33 |  | 0.312 | 1.22 | 200.72 |
| sIL-1RII | 75.70 | 111.1 | 91.29 |  | 0.430 | 0.574 | 87.48 |
| PDGF-BB | 107.0 | 109.6 | 18.52 |  | 0.609 | 0.845 | 105.41 |
| sE-Selectin | 67.90 | 102.7 | 91.72 |  | 0.325 | 1.07 | 190.48 |
| Bcl-2 | 76.90 | 100.8 | 67.12 |  | 0.445 | 0.811 | 152.21 |
| HSP90a | 54.80 | 93.30 | 127.39 |  | 0.253 | 0.482 | 126.31 |
| HSP60 | 37.60 | 90.77 | 116.22 |  | 0.298 | 0.515 | 101.28 |
| CXCL9/MIG | 54.90 | 88.78 | 123.03 |  | 0.286 | 0.466 | 144.37 |
| SDF-1a+B | 46.10 | 81.83 | 104.59 |  | 0.309 | 0.510 | 127.54 |
| IGF-1R | 49.40 | 69.29 | 90.55 |  | 0.279 | 0.474 | 151.83 |
| PAI-1 | 60.20 | 65.64 | 55.99 |  | 0.299 | 0.418 | 97.91 |
| Amphiregulin | 51.80 | 57.90 | 57.94 |  | 0.294 | 0.324 | 52.39 |
| MIP-1d | 43.10 | 56.87 | 77.42 |  | 0.274 | 0.351 | 94.62 |
| MMP-3 | 43.50 | 51.75 | 63.38 |  | 0.233 | 0.319 | 117.77 |
| AGRP | 6.71 | 50.86 | 236.94 |  | 0.045 | 0.281 | 249.97 |
| OPG | 35.60 | 50.70 | 76.75 |  | 0.215 | 0.396 | 164.06 |
| TSLP | 32.30 | 48.70 | 96.39 |  | 0.178 | 0.385 | 191.29 |
| CCL14a/HCC-1 | 29.50 | 42.76 | 100.70 |  | 0.127 | 0.205 | 106.66 |
| IL-20 | 36.10 | 41.78 | 44.04 |  | 0.217 | 0.408 | 194.11 |
| IL-11 | 38.70 | 40.91 | 55.12 |  | 0.196 | 0.242 | 84.49 |
| Fractalkine | 33.80 | 40.38 | 74.49 |  | 0.188 | 0.232 | 84.38 |
| sFas | 38.50 | 39.63 | 33.81 |  | 0.094 | 0.100 | 95.47 |
| FGF-19 | 39.30 | 39.05 | 9.15 |  | 0.185 | 0.301 | 122.71 |
| sVEGFR3 | 37.30 | 37.69 | 13.00 |  | 0.183 | 0.283 | 119.14 |
| MIP-4 | 18.60 | 36.72 | 279.81 |  | 0.089 | 0.183 | 321.26 |
| sIL-4R | 10.80 | 32.35 | 137.05 |  | 0.035 | 0.397 | 301.91 |
| CNTF | 30.70 | 32.07 | 31.12 |  | 0.176 | 0.245 | 107.14 |
| FGF-21 | 3.93 | 31.87 | 152.49 |  | 0.029 | 0.151 | 153.59 |
| sVEGFR1 | 29.30 | 30.55 | 24.98 |  | 0.156 | 0.217 | 104.36 |
| MICA | 21.20 | 29.09 | 103.75 |  | 0.085 | 0.295 | 217.41 |
| IL-29 | 24.00 | 24.34 | 8.61 |  | 0.115 | 0.180 | 114.10 |
| NT-Pro-BNP | 21.00 | 24.21 | 114.51 |  | 0.094 | 0.161 | 141.06 |
| Eotaxin-2 | 18.40 | 22.75 | 181.96 |  | 0.097 | 0.122 | 192.49 |
| IL-33 | 14.10 | 20.85 | 93.53 |  | 0.072 | 0.229 | 303.29 |
| PYY | 10.40 | 19.61 | 83.16 |  | 0.073 | 0.142 | 158.97 |
| RANKL | 12.60 | 17.23 | 82.63 |  | 0.073 | 0.118 | 161.07 |
| XCL1/Lymphotactin | 14.40 | 15.76 | 38.71 |  | 0.074 | 0.133 | 192.32 |
| AFP | 15.50 | 15.64 | 16.69 |  | 0.074 | 0.120 | 119.58 |
| MMP-13 | 9.97 | 12.57 | 58.87 |  | 0.053 | 0.095 | 123.73 |
| MMP-1 | 6.37 | 11.89 | 236.07 |  | 0.033 | 0.074 | 272.47 |
| PDGF-AB/BB | 11.30 | 11.46 | 16.94 |  | 0.064 | 0.092 | 112.43 |
| CXCL7/NAP2 | 8.93 | 10.94 | 129.62 |  | 0.045 | 0.078 | 142.87 |
| 6CKine | 10.10 | 10.67 | 29.29 |  | 0.061 | 0.082 | 95.72 |
| sCD137/4-1BB | 8.51 | 10.26 | 72.72 |  | 0.037 | 0.049 | 92.91 |
| Eotaxin-3 | 10.10 | 10.17 | 20.59 |  | 0.054 | 0.080 | 113.12 |
| PTH | 6.06 | 10.08 | 98.91 |  | 0.034 | 0.103 | 233.60 |
| Glucagon | 9.03 | 10.00 | 49.90 |  | 0.046 | 0.069 | 106.78 |
| GLP-1 | 9.89 | 9.71 | 16.89 |  | 0.045 | 0.079 | 133.40 |
| Prolactin | 6.90 | 9.57 | 76.17 |  | 0.046 | 0.066 | 98.38 |
| TRAIL | 5.59 | 9.52 | 109.70 |  | 0.035 | 0.056 | 112.18 |
| IL-28A | 6.44 | 9.42 | 61.43 |  | 0.041 | 0.086 | 160.00 |
| Tenascin C | 8.68 | 9.25 | 92.19 |  | 0.043 | 0.077 | 134.76 |
| ENA-78 | 8.73 | 9.05 | 23.02 |  | 0.049 | 0.074 | 126.62 |
| Betacellulin | 6.27 | 9.04 | 219.02 |  | 0.039 | 0.064 | 209.11 |
| CD-105 | 7.89 | 8.24 | 22.45 |  | 0.038 | 0.064 | 125.68 |
| LIF | 7.86 | 8.05 | 16.69 |  | 0.044 | 0.064 | 111.33 |
| Flt-3 Ligand | 5.32 | 7.72 | 91.03 |  | 0.031 | 0.044 | 93.94 |
| RANTES | 4.14 | 7.35 | 114.19 |  | 0.030 | 0.041 | 97.01 |
| EPCAM | 6.23 | 7.26 | 75.51 |  | 0.031 | 0.056 | 132.65 |
| Granzyme A | 1.11 | 7.06 | 249.11 |  | 0.005 | 0.038 | 221.97 |
| sCD30 | 6.43 | 6.62 | 17.11 |  | 0.032 | 0.050 | 117.65 |
| IL-21 | 5.55 | 6.29 | 29.89 |  | 0.033 | 0.055 | 142.65 |
| Amylin | 5.93 | 5.93 | 31.45 |  | 0.026 | 0.043 | 115.14 |
| PP | 5.36 | 5.79 | 34.58 |  | 0.027 | 0.042 | 111.83 |
| CXCL6/GCP2 | 4.15 | 5.73 | 99.81 |  | 0.018 | 0.044 | 143.37 |
| VEGF | 4.85 | 5.54 | 69.89 |  | 0.019 | 0.046 | 140.63 |
| GH | 3.77 | 5.53 | 87.08 |  | 0.023 | 0.035 | 97.88 |
| IL-8 | 0.817 | 5.44 | 181.18 |  | 0.005 | 0.029 | 210.35 |
| SCF | 5.42 | 5.43 | 36.41 |  | 0.031 | 0.035 | 67.74 |
| Progesterone Receptor | 4.69 | 5.28 | 69.87 |  | 0.024 | 0.030 | 112.08 |
| ACTH | 3.97 | 5.28 | 73.74 |  | 0.031 | 0.094 | 159.17 |
| MCP-4 | 4.67 | 5.06 | 60.75 |  | 0.027 | 0.038 | 98.98 |
| PIGF | 4.36 | 4.88 | 61.87 |  | 0.023 | 0.031 | 82.04 |
| NGF | 3.52 | 4.05 | 70.42 |  | 0.016 | 0.032 | 139.41 |
| tPAI-1 | 3.47 | 3.73 | 32.27 |  | 0.012 | 0.015 | 104.50 |
| Ghrelin | 3.34 | 3.35 | 17.81 |  | 0.016 | 0.027 | 132.14 |
| IL-16 | 3.19 | 3.35 | 31.09 |  | 0.018 | 0.026 | 104.31 |
| TIMP-4 | 2.27 | 3.16 | 72.16 |  | 0.013 | 0.019 | 95.42 |
| CCL19/MIP3β | 2.78 | 3.07 | 23.24 |  | 0.016 | 0.026 | 149.50 |
| sFasL | 2.74 | 3.05 | 44.10 |  | 0.014 | 0.019 | 100.37 |
| Oncostatin | 2.53 | 2.64 | 24.41 |  | 0.012 | 0.019 | 112.78 |
| sRAGE | 2.24 | 2.63 | 47.84 |  | 0.011 | 0.024 | 223.58 |
| Epiregulin | 2.18 | 2.58 | 77.99 |  | 0.012 | 0.020 | 128.45 |
| sCD40L | 1.84 | 2.36 | 141.35 |  | 0.009 | 0.018 | 170.02 |
| sIL-1RI | 2.18 | 2.20 | 15.39 |  | 0.011 | 0.016 | 115.01 |
| MMP-12 | 1.84 | 2.09 | 39.88 |  | 0.009 | 0.017 | 126.35 |
| CXCL11/I-TAC | 1.48 | 1.84 | 81.93 |  | 0.008 | 0.012 | 132.36 |
| CTACK | 1.36 | 1.74 | 138.44 |  | 0.007 | 0.024 | 440.04 |
| MCP-2 | 1.64 | 1.72 | 24.62 |  | 0.009 | 0.013 | 91.84 |
| IL-6 | 0.730 | 1.62 | 134.56 |  | 0.005 | 0.009 | 139.65 |
| BDNF | 1.49 | 1.56 | 23.54 |  | 0.008 | 0.011 | 92.68 |
| I-309 | 0.935 | 0.949 | 16.49 |  | 0.005 | 0.007 | 97.88 |
| CCL20/MIP3α | 0.773 | 0.865 | 50.90 |  | 0.004 | 0.007 | 157.50 |
| BCA-1/CXCL13 | 0.624 | 0.701 | 56.39 |  | 0.004 | 0.005 | 87.93 |
| INF-b | 0.442 | 0.647 | 95.51 |  | 0.002 | 0.005 | 172.03 |
| GIP | 0.507 | 0.527 | 40.57 |  | 0.003 | 0.004 | 105.76 |
| Granzyme B | 0.417 | 0.460 | 53.31 |  | 0.002 | 0.003 | 110.83 |
| TARC | 0.403 | 0.444 | 54.99 |  | 0.002 | 0.003 | 111.41 |
| Perforin | 0.198 | 0.239 | 80.53 |  | 0.001 | 0.002 | 114.10 |
| INF-ω | 0.166 | 0.175 | 18.03 |  | 0.001 | 0.001 | 121.90 |
| IL-1b | 0.154 | 0.162 | 26.59 |  | 0.001 | 0.001 | 139.58 |
| TNFα | 0.118 | 0.128 | 41.00 |  | 0.001 | 0.001 | 108.72 |
| TSH^‡^ | 0.050 | 0.099 | 170.58 |  | 0.0003 | 0.0007 | 216.20 |
| CA 15-3 ^‡^ | 9.01 | 15.99 | 125.98 |  | 0.0343 | 0.0839 | 146.53 |
| CA-125 ^‡^ | 1.64 | 9.39 | 389.06 |  | 0.0104 | 0.0522 | 393.48 |
| CA 19-9 ^‡^ | 1.09 | 5.70 | 740.57 |  | 0.0069 | 0.0396 | 748.26 |
| Phospho-HSP27 ^‡^ | 0.045 | 0.342 | 287.62 |  | 0.0003 | 0.0020 | 317.73 |
| CA72-4 ^‡^ | 0.116 | 0.147 | 131.33 |  | 0.0006 | 0.0010 | 146.62 |
| Tg II ^‡^ | 0.104 | 0.106 | 24.38 |  | 0.0005 | 0.0008 | 115.60 |
| FSH ^‡^ | 6.63 | 15.27 | 201.34 |  | 0.0448 | 0.1244 | 259.44 |
| LH ^‡^ | 3.98 | 7.94 | 155.19 |  | 0.0173 | 0.0359 | 147.59 |
| †Values normalized based on urinary creatinine measurements; absolute biomarker concentration (pg/ml)/creatinine concentration (mg/dl)  ‡Absolute concentrations measured in U/ml | | | | | | | |
